# A Fœtus at Full Period, without Investing Membranes or Liquor Amnii

**Published:** 1857-05

**Authors:** J. P. H. Stevenson

**Affiliations:** Robinson, Ill.


ARTICLE II.—*A Fœtus at Full Period, without Investing Membranes or Liquor Amnii.* By J. P. H. STEVENSON, of Robinson, Ill.

An obstetrical case, presenting some points of interest, recently came under my care, and I concluded to communicate it to the profession through the pages of your valuable *Journal.*

I was called, February 24, 1857, to Mrs. M———, aged twenty-nine years, in labor three hours with her fourth child. She had gone to full term, and I found the pains characteristic of the first stage of labor. I interrogated her in regard to the rupture of the membranes and discharge of the waters, and she informed me, that it had not occurred. On an examination per *vaginum*, I found the *os uteri* within reach and dilated to about the size of a twenty-five cent piece, and quite dilatable. The vertex presented, and it was evident to me that there were no membranes interposing between my finger and the fœtal cranium. I again interrogated her in regard to the discharge of the waters, and she still insisted that it had not occurred. I used the precaution to examine her clothing, to ascertain if they were wet from the discharge, but found them dry. I accounted at that time for the absence of the membranes in this way, that the vertex had ruptured them, and they had receded so as not to be in reach at the time of the examination, and adhered so closely to the cranium that the liquor *amnii* could not escape. The labor proved a lingering one lasting thirty hours, at the end of which time I delivered her of a very plump female child, whose weight was about eight pounds, but still no appearance of membranes or liquor *amnii*. As soon as I could sever the cord, and before the cavity of the womb became filled with blood, I introduced my hand to ascertain if the membranes were not adhering to its walls, but they were not to be found. As no portion of the *placenta* had been separated from its maternal attachment, I found the interior of the womb quite dry, and covered to the depth of a sixteenth of an inch, as was the body of the child, with a white, tenacious, cheesy-like material. That which covered the body of the child, adhered so closely that it could not be removed by ordinary washing. I took the pains to separate gently, with my fingers, a portion of this internal coating and examine it before I separated the *placenta*. I then separated the *placenta* and examined it, but found no trace of membranes adhering to its walls. It was very certain that no liquor *amnii* was to be discovered at birth. The lady, being an intelligent woman, asked the reason of this strange occurrence, and remarked, after the labor, that her clothing was quite dry. In her third accouchment the waters were so abundant I was compelled to *puncture* the membranes early in the labor. On the third day she had inflammation of the womb, caused no doubt by that tenacious substance covering the interior and acting as a foreign body. It was promptly controlled by calomel, opium, injections, and fomentations. At this time mother and child are both well.

This case may appear as strange to the profession as it did to me when it came under my observation, for the reason that it comes in direct contact with our preconceived notions and theories on the subject. I do not remember, in the course of my reading to have noticed the mention of such a case. In *Meigs Obstetrics*, page 189, I find the following very emphatic language on this subject, speaking of Pregnancy, he says:—

'Suffice it for me to say, that, at any time in the course of the whole career, that career may, by the physician, be instantly arrested and brought to a speedy close by discharging or withdrawing the ovum, or by taking away the retentive power of the survex uterii; for to discharge the waters of the *amnion* by *puncture*, or to dilate the canal of the survex with a sponge-tent, or to energize immoderately the facultus expultrix of the *fundus* and *corpus* uteri by means of ergot, is to arrest and bring to a close the whole operation of the reproductive process.'

Thus we have the indirect testimony of one of our best authors to the universal presence of the membranes and liquor amnii, and the positive assertion, that, in the event of a breach of continuity in the membranes, inevitable destruction to the products of conception. But a question here arises, may not the membranes and the liquor amnii in the last days of pregnancy, when the process of development is carried on so rapidly, be absorbed, and they being absorbed, may not the fœtus remain alive in utero until full term. Let our hypothesis on this point be as it may, the above case furnishes evidence that, at least, one fœtus has lived in utero, without either membranes or liquor amnii to be found at birth: but how long it may have lived without these necessary appendages, 'this deponent saith not.' I say necessary appendages; for, without them, every labor otherwise perfectly natural would be converted into a preternatural one.

A German medical journal recently mentioned a case of the total absence of the umbilical cord at full term.

